# Heterogeneous Polymer Dynamics Explored Using Static ^1^H NMR Spectra

**DOI:** 10.3390/ijms21155176

**Published:** 2020-07-22

**Authors:** Todd M. Alam, Joshua P. Allers, Brad H. Jones

**Affiliations:** Department of Organic Materials Science, Sandia National Laboratories, Albuquerque, NM 87185, USA; jpaller@sandia.gov (J.P.A.); bhjones@sandia.gov (B.H.J.)

**Keywords:** NMR, polymers, chain dynamics, second moment

## Abstract

NMR spectroscopy continues to provide important molecular level details of dynamics in different polymer materials, ranging from rubbers to highly crosslinked composites. It has been argued that thermoset polymers containing dynamic and chemical heterogeneities can be fully cured at temperatures well below the final glass transition temperature (T_g_). In this paper, we described the use of static solid-state ^1^H NMR spectroscopy to measure the activation of different chain dynamics as a function of temperature. Near T_g_, increasing polymer segmental chain fluctuations lead to dynamic averaging of the local homonuclear proton-proton (^1^H-^1^H) dipolar couplings, as reflected in the reduction of the NMR line shape second moment (*M*_2_) when motions are faster than the magnitude of the dipolar coupling. In general, for polymer systems, distributions in the dynamic correlation times are commonly expected. To help identify the limitations and pitfalls of *M*_2_ analyses, the impact of activation energy or, equivalently, correlation time distributions, on the analysis of ^1^H NMR *M*_2_ temperature variations is explored. It is shown by using normalized reference curves that the distributions in dynamic activation energies can be measured from the *M*_2_ temperature behavior. An example of the *M*_2_ analysis for a series of thermosetting polymers with systematically varied dynamic heterogeneity is presented and discussed.

## 1. Introduction

Solid-state NMR spectroscopy remains an important tool for the characterization of the structure and dynamics in a wide range of materials [1,2,3,4,5]. Improvements in magic angle spinning (MAS) spinning speeds; advances in heteronuclear, multiple dimensional, and multiple quantum NMR pulse sequences; plus the sensitivity gains realized from dynamic nuclear polarization (DNP) methods continue to impact the use of NMR to probe a variety of different material phenomena. While many of these advances help resolve additional molecular level details, there is a corresponding increase in the complexity of implementation. One of the oldest and, perhaps, simplest methods for the analysis of NMR spectra involves measuring the second moment (*M*_2_) of the line shapes as a function of the temperature or composition [6,7] and has proven to be indispensable in characterizing dynamics and local structures in amorphous and disordered materials. Static (non-spinning) NMR line shapes are broadened by homonuclear and heteronuclear dipolar couplings between different nuclei, quadrupolar couplings (for spin *I* > ½), chemical shielding anisotropy, or chemical shift dispersions, thus providing a handle to probe local structures and dynamics. For example, ^7^Li, ^23^Na, ^31^P, and ^133^Cs NMR *M*_2_ results have been reported for glasses in analyses of structures and ion dynamics [8,9,10,11,12], as well as ^1^H NMR *M*_2_ studies of dynamics, miscibility, and chain orientations in polymers [13,14,15,16,17,18,19,20]. While static NMR is hampered by low spectral resolution, it does benefit from being sensitive to local motions on the order of the line width and can be employed over very wide temperatures ranges.

**Scheme 1:**
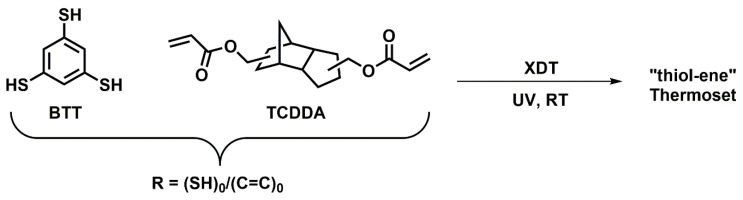
Chemical structures of the 1,3,5-benzenetrithiol (BTT) and tricyclodecane dimethanol diacrylate (TCDDA) monomers used in the preparation of high-transition temperature (T_g_) thiol-acrylate networks through a room-temperature (RT) ultraviolet (UV) cure using the *p*-xylylene bis(*N,N*-diethyldithiocarbamate) (XDT) photo-initiator, where variations in the ratio R control the network heterogeneity.

Our group has recently explored the impact of dynamic heterogeneity on polymer thermosets that can be cured (polymerized) at temperatures well below the final observed glass transition temperature T_g_ [20]. Here dynamic heterogeneity refers to the existence of regions within the thermoset where the polymer chain segment fluctuations have different correlation times or exhibit differences in the widths of the correlation time distributions. NMR proves to be a powerful tool to probe these heterogeneities at the molecular level. Thiol–ene polymerizations combine both chain-growth and step-growth mechanisms, enabling the systematic variation of dynamic heterogeneity through the stoichiometry of the polymerization mixture [21,22,23,24,25,26,27,28], which can be used to control the relationship between the cure temperature and the final T_g_. We prepared a series of high-T_g_ materials, in which the heterogeneity was modulated by the reacting mixtures of the aromatic monomer 1,3,5-benzenetrithiol (BTT) and tricyclodecane dimethanol diacrylate (TCDDA) with differing ratios of the reactive functional groups. These mixtures are identified by R = (SH)_0_/(C=C)_0_, where (SH)_0_ and (C= C)_0_ were the initial concentrations of the thiol and acrylate functionality, respectively. The polymer mixtures were ultraviolet (UV) cured in the presence of the photo-initiator *p*-xylylene bis(*N,N*-diethyldithiocarbamate) (XDT), as shown in Scheme 1. The resulting thermosets incorporate both the chain-growth homo-polymerization of the acrylate groups and step-growth co-polymerization of the thiol and acrylate groups, which yield comparatively heterogeneous and homogeneous networks, respectively. Chain-growth thermosets are described as containing a nonuniform distribution of crosslinks at the nanoscale [29], which should be reflected in the nonuniform correlation time (or relaxation rates) distributions for the polymer chain fluctuations. By decreasing R (lower thiol concentration), the polymerization is biased towards the chain-growth mechanism and is expected to increase the heterogeneity in the local crosslink density and, correspondingly, an increase in the distribution of the polymer chain relaxation rates. Additional physical characterizations of these TCDDA-BTT networks have previously been reported [20]. In that study, we reported the temperature variation of the second moment (*M*_2_) of the ^1^H NMR spectral line shape, along with qualitative arguments for increased dynamic heterogeneity in low R value networks. In the current study, we present an analysis of how dynamic distributions or dynamic heterogeneity are reflected in both the *M*_2_ temperature variation and the estimated polymer chain correlation times (τ_c_).

## 2. Results and Discussion

**Figure 1:**
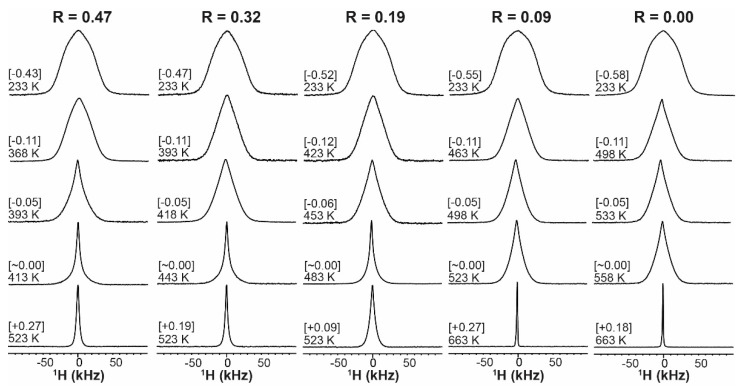
Static solid-state ^1^H NMR spectra as a function of select sample temperature for the ultraviolet (UV)-cured 1,3,5-benzenetrithiol-tricyclodecane dimethanol diacrylate (BTT-TCDDA) polymer networks of varying thiol-acrylate stoichiometry (R = (SH)_0_/(C=C)_0_). The reduced temperatures (T-T_g_)/T_g_ are shown in square brackets, with the transition temperature (T_g_) occurring at (T-T_g_)/T_g_ = 0. Narrowing of the NMR line width is observed for temperatures above the polymer glass transition temperature T_g_. The middle three rows are at equivalent reduced temperatures, while the upper and lower rows show the lowest and highest temperatures probed for that stoichiometry, respectively.

Static solid-state ^1^H NMR spectra for the UV-cured BTT-TCDDA networks as a function of temperature and R are shown in Figure 1. At 233 K (−40 °C), a single unresolved broad resonance having a full width at a half-maximum line width of~50 kHz is observed for all R ratios. This broad resonance originates from the strong homonuclear ^1^H-^1^H dipolar couplings present in these rigid, glassy polymers. At these low temperatures, the local polymer chain dynamics are slow compared to the timescale of the dipolar interaction (i.e., 1/τ_c_ << 2π(*M*_2_)^1/2^, where τ_c_ is the correlation time of the chain fluctuations and (*M*_2_)^1/2^ is related to the spectral line width). Note, for the solid-state ^1^H NMR spectra at low temperatures, there are no narrow spectral components overlapping the broad resonances. This lack of a narrow resonance clearly shows that highly mobile polymer fractions (1/τ_c_ >> 2π(*M*_2_)^1/2^) from comparatively low crosslink density regions are not present. Another way to describe this is that, if there are low crosslink density regions, they must have similar dynamic responses to the rest of the network when well below the T_g_. With increasing temperatures, additional polymer dynamics are activated, leading to the motional averaging of the dipolar coupling (1/τ_c_~2π(*M*^2^)^1/2^) and gradual narrowing of the NMR line resonance. This motional averaging dramatically increases near the T_g_, where the rate and magnitude of the polymer chain fluctuations (α-relaxation) become large enough to ultimately produce the fully dynamically averaged narrow line shapes observed at high temperatures (Figure 1). With the decreasing R, the temperature at which the α-relaxation becomes activated increases and mirrors the change observed in the dynamic mechanical analysis (DMA) (Supplementary Materials Figure S1). For the BTT-TCDDA networks discussed here, the dynamic heterogeneities (i.e., a distinct mixture of narrow and broad resonances) observed in the ^1^H NMR line shape as a function of R near the T_g_ are not as pronounced as those previously noted in related networks [20] and most likely reflect the differences in the UV light source intensity employed and the actual sample temperature during the cure process.

While it is common during the analysis of static ^1^H NMR line shapes to simply deconvolute the resonance into mobile and immobile fractions, polymer chain dynamics realistically involve a distribution of motional rates, leading to a complex superposition of different motionally averaged line shapes. Here, we describe an analysis of *M*_2_ calculated using the spectral intensity at frequency ω over the entire line shape for each NMR spectrum using (see Appendix A for details):

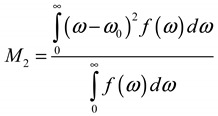
(1)


**Figure 2:**
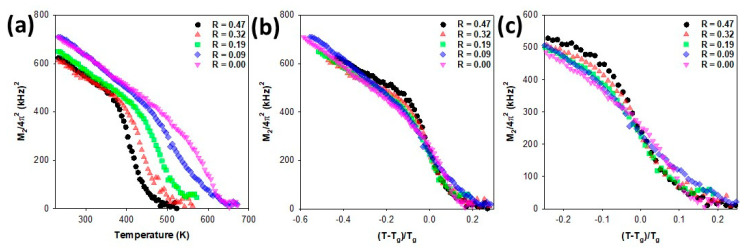
Static solid-state ^1^H NMR spectral second moment (M_2_) for BTT-TCDDA networks with different thiol-acrylate stoichiometry (R = (SH)_0_/(C=C)_0_) as a function of (**a**) the sample temperature, (**b**) the reduced temperature, and (**c**) the reduced temperature expanded near the T_g_ for each composition.

The temperature variation of the ^1^H NMR *M*_2_ for BTT-TCDDA networks as a function of R is shown in Figure 2a. In the low temperature regime *M*_2_ for R = 0.00 and 0.09, the networks are similar but are significantly larger than the *M*_2_ for R = 0.47 and 0.32, while R = 0.19 is intermediate between these extremes. The larger *M*_2_ at a low temperature (increased magnitude of the ^1^H-^1^H dipolar coupling) may result from multiple factors. The first possibility is that the TCDDA component has an increased intra- or intermolecular dipolar coupling (higher intra- or intermolecular proton density) than the BTT and that higher TCDDA concentrations (decreasing R) will therefore increase the *M*_2_. The proton density for the pure monomers is estimated to be 0.087 moles H/cm^3^ and 0.047 moles H/cm^3^ for BTT and TCDDA, respectively, supporting the argument that proton density increases with decreasing R. While the *M*_2_ has not been theoretically calculated for the cured TCDDA-BTT polymers, as the network structures are not known, we argue that the 100-kHz^2^ differences in the *M*_2_ at 233 K cannot be fully explained by the compositional change. The most reasonable explanation is that, at this temperature, the TCCDA rich compositions have dynamics that are more representative of the rigid glassy state, since, at 233 K, the temperature is further from their respective T_g_ values. To support this, the *M*_2_ is plotted with respect to the scaled temperature (T-T_g_)/T_g_, as shown in Figure 2b, which reveals that all compositions trend to the same *M*_2_ limit. Due to probe hardware limitations, we were not able to investigate temperatures below 233K, preventing us from reaching the rigid lattice limit for all compositions.

Between 233 K and 400 K, there is a gradual decrease in the *M*_2_ with the increasing temperature (Figure 2a), which is attributed to the β-relaxation process involving local, noncooperative dynamics in the BTT-TCDDA network. The *M*_2_ temperature variation for this β-relaxation process is similar for all compositions. As the temperature is further increased through a given T_g_, the respective *M*_2_ rapidly decreases due to dynamic averaging as the polymer chain motions become faster and more dominant (α-relaxation), until finally reaching the rubbery plateau regions for T > T_g_. The expansion of the *M*_2_ temperature variation for the BTT-TCDDA networks at reduced temperatures near (T-T_g_)/T_g_~0 (Figure 2c) reveals distinctly different dynamics, which we will attribute to differences in the activation energy (and corresponding correlation times) for the polymer chain motions in subsequent sections. Additional NMR relaxation experiments, including spin lattice relaxation (T_1_) and spin-spin relaxation (T_2_), were not pursued for the current study due to the desire to match the NMR heating/cooling rates to the heating/cooling rates of the DMA analysis as closely as possible.

### 2.1. Impact of Activation Energy on M_2_

**Figure 3:**
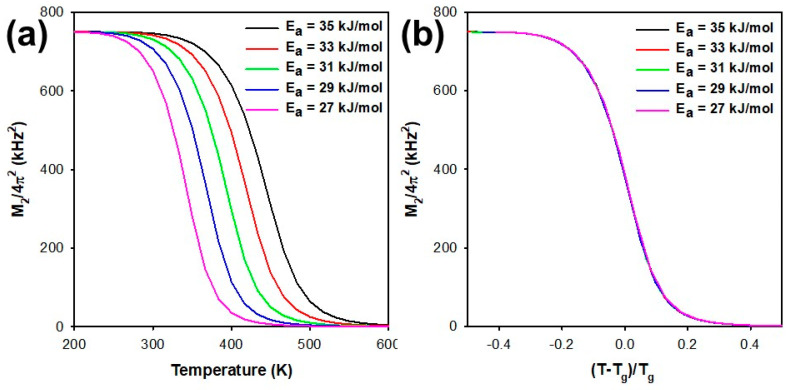
Simulated variation of the spectral second moment (*M*_2_) calculated from Equation (1) for the different dynamic activation energies (E_a_) as a function of the (**a**) temperature and (**b**) reduced temperature.

To extract additional information regarding polymer chain dynamics from the ^1^H NMR spectra, it is instructive to model the effect of different variables on the *M*_2_ behavior. Figure 3 shows simulations of the *M*_2_ temperature variation for varying activation energies (E_a_) of the dynamic process leading to the motional averaging. The temperature behavior of the *M*_2_ is defined using Equation (A5) (additional discussion provided in the Appendix A), where we have assumed an Arrhenius behavior for the correlation times τ_c_ using:

(2) $$\tau_{c}\left(T\right)=\tau_{0}\exp\left[E_{a}/R_{gas}T\right]$$

where τ_0_ is the pre-exponential correlation time, E_a_ the activation energy, and *R_gas_* is the gas constant. The pre-exponential term was fixed at τ_0_ = 0.5905 ns for all simulations, unless otherwise noted. This τ_0_ value is in the middle of the range experimentally determined (see Table 1 and later discussion). An example of the simulated *M*_2_ variations for changing τ_0_ is shown in Figure S2. A more complicated non-Arrhenius temperature behavior of τ_c_, such as the Vogel-Fulcher-Tammann (VFT) relationship [30,31,32], could also be considered. As expected, the transition from the rigid lattice *M*_2_ limit to the fully motionally averaged *M*_2_ occurs at higher temperatures with increasing E_a_. The *M*_2_ temperature variations for this range of E_a_ overlap when scaled to the reduced temperature (T-T_g_)/T_g_, as shown in Figure 3b, with this scaling behavior being well-known in the analysis of polymer relaxation processes [33,34,35].

### 2.2. Distributions in Dynamic Rates

**Figure 4:**
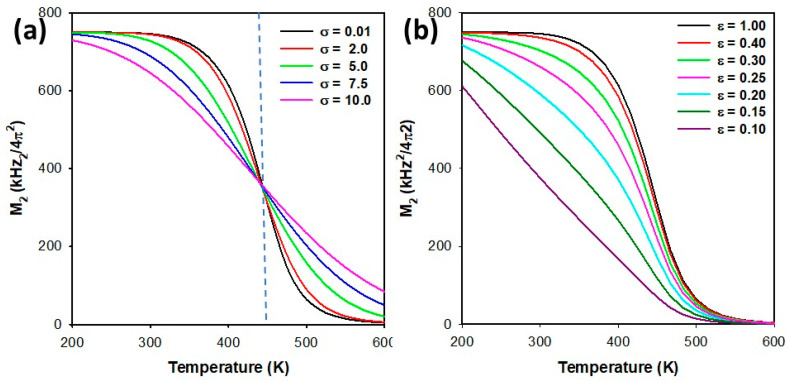
Simulated temperature variation of the spectral second moment (*M*_2_) using Equation (1) for different distribution widths, assuming (**a**) a Gaussian distribution in E_a_ (using Equation (A8)) and (**b**) a Davidson-Cole distribution (using Equation (A9)) of correlation times. Simulations assume a constant mean activation energy of 33 kJ/mol.

In amorphous polymers, it is expected that the dynamics include a distribution of chain relaxation correlation times, as previously included in NMR line shapes and relaxation time analyses [36,37,38]. Since a variation in E_a_ moves the temperature at which the T_g_ transition is observed (Figure 3), the presence of a distribution of activation energies will therefore impact the *M*_2_ temperature variation. As an example, Figure 4a shows the simulated *M*_2_ variation with the introduction of a Gaussian distribution of the log mean correlation time τ * or, equivalently, a Gaussian distribution of E_a_ (see Equation (A8)) with different standard deviations σ. Examples of these Gaussian distributions are shown in Figure S3. The results are labeled as a function of σ(E_a_) for direct comparison to Figure 3. With the increasing σ, the rate of change of the *M*_2_ with the temperature is reduced, with the width of the T_g_ transition broadened, and is reminiscent of the DMA results shown in Figure S1. This broadening behavior results from the increasing fraction of polymer chains having either slower (higher E_a_) or faster (lower E_a_) relaxation rates. For a Gaussian distribution, the *M*_2_ intensities at different σ are equivalent at the same T_g_ temperature (here, 442 K), because the mean E_a_ is independent of σ for a symmetric distribution.

**Figure 5:**
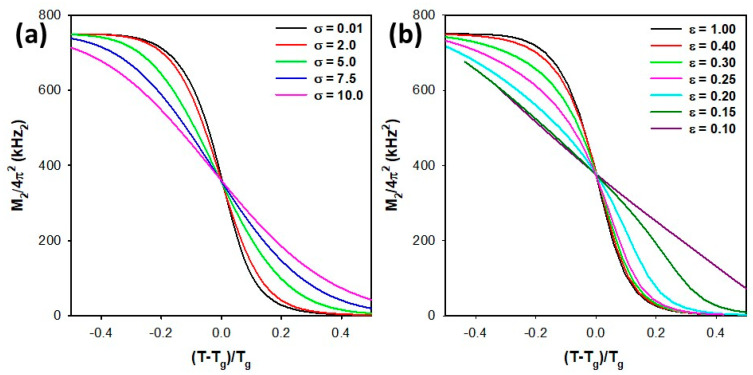
Simulated variation of the NMR second moment (*M*_2_) as a function of the reduced temperature for the (**a**) Gaussian E_a_ distribution described by Equation (A8) and (**b**) a Davidson-Cole correlation time distribution described by Equation A9.

Another distribution commonly employed to describe polymer relaxation is the Davidson-Cole (DC) relationship [39] defined by Equation (A9), with an example of the distribution shown in Figure S3. The temperature variation of the *M*_2_ as a function of the increasing distribution breadth (decreasing ε) is shown in Figure 4b. The behavior is distinct from that observed for a Gaussian distribution. In the DC distribution model, the *M*_2_ transition is broadened with a decreasing ε for temperatures below the original T_g_ (transition temperature). This broadening results from a larger fraction of polymer chain motions being activated at lower temperatures (1/τ_c_~2π(*M*^2^)^1/2^) due to the increased concentration of regions (e.g., defects, reduced crosslink density, etc.) having reduced E_a_ values. For temperatures above the original T_g_ transition, there is limited impact on the *M*_2_ behavior, because the DC distribution (Equation (A9)) is described by the maximum correlation time $\left(\tau_{c}^{0}\right)$ above which the probability of the slower dynamic processes vanishes. The asymmetry of the DC probability shifts the observed T_g_ (defined here as the midpoint in the *M*_2_ transition) to lower temperatures. The variations in the *M*_2_ as a function of the reduced temperatures for systems with either a Gaussian or a DC distribution are shown in Figure 5a,b, respectively. For small distribution widths, the *M*_2_ temperature behavior is very similar and only begins to deviate for large distributions.

**Figure 6:**
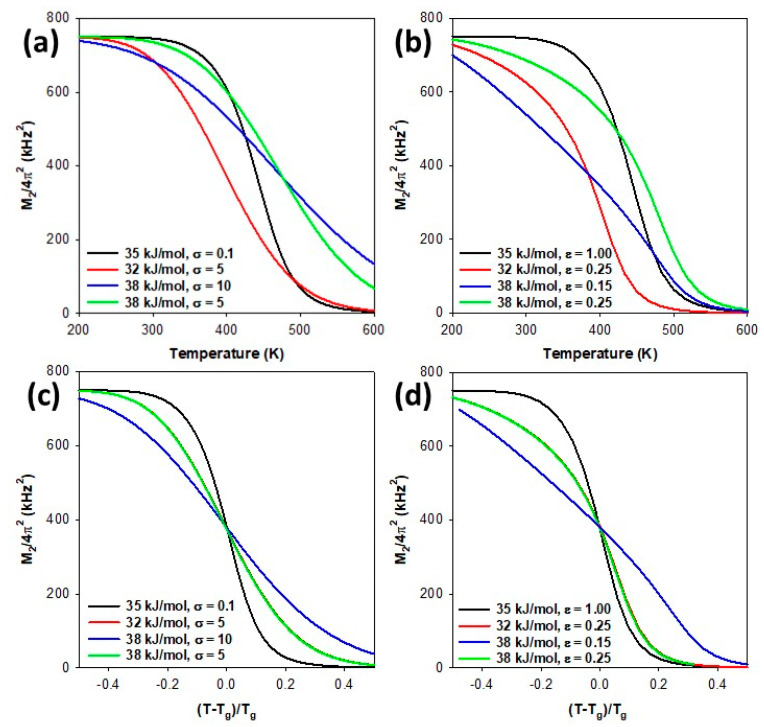
Simulated variation of the spectral second moment (*M*_2_) as a function of the temperature and reduced temperature for the (**a**,**c**) Gaussian E_a_ distribution and (**b**,**d**) a Davidson-Cole correlation time distribution with different mean E_a_ and distribution widths.

The comparison of the *M*_2_ temperature variation between different network compositions may be complicated by differences in both the E_a_ (different T_g_ values) and distribution widths. This issue is explored in Figure 6 for the Gaussian and DC distributions. The *M*_2_ temperature behavior (Figure 6a,b) does not readily allow differences in the distribution to be realized, but, by simply mapping the *M*_2_ behavior to a reduced temperature scale, the presence of the distributions is clear, regardless of the different E_a_ (Figure 6c,d). The *M*_2_ behavior is related to the relative magnitude of σ with respect to the E_a_, but for similar ranges of the E_a_, the *M*_2_ curves overlap well for a given value of σ. Therefore, by plotting the *M*_2_ with respect to reduced temperatures, it should be possible to experimentally measure distributions of polymer chain dynamics near T_g_ from static solid-state ^1^H NMR experiments.

### 2.3. Multiple Dynamic Processes

**Figure 7:**
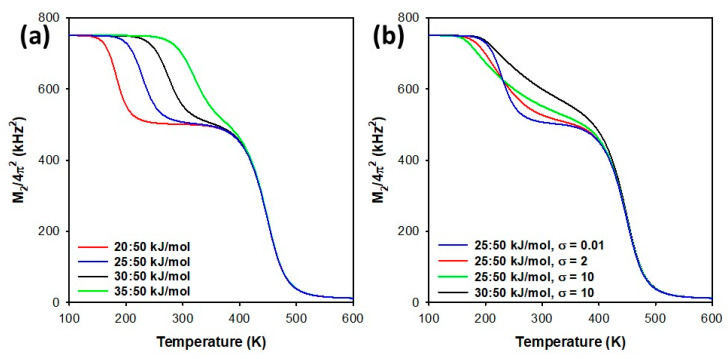
Simulated variation of the spectral second moment (*M*_2_) as a function of the temperature for two dynamic process (Equation A10) defined by the activation energies E_a_(1):E_a_(2) as a function of (**a**) different E_a_(1) values and (**b**) as a function of varying E_a_(1) distribution widths. The distribution of E_a_(2) was fixed at σ = 0.01.

The impact of multiple dynamic processes on the *M*_2_ temperature behavior has previously been discussed by several authors [40,41,42,43,44,45,46,47,48]. For two noncorrelated dynamic processes, the *M*_2_ behavior is defined by Equation (A10). An example is shown in Figure 7 involving a slow dynamic process at lower temperatures (β-relaxation), defined by the activation energy E_a_(1), and a second dynamic process (α-relaxation, glass transition) at higher temperatures defined by the activation energy E_a_(2). In these simulations, it is very easy to distinguish the two separate dynamic averaging transitions (Figure 7a) by the two-step features that are predicted, consistent with the work of Bilski and coworkers [41]. The addition of E_a_(1) distributions to the β-relaxation transition (Figure 7b) begins to obscure the reduction step in the *M*_2_ resulting from this dynamic process. This *M*_2_ behavior is reminiscent of the *M*_2_ variation seen before the main T_g_ transition for the BTT-TCDDA networks (Figure 2). We were unable to reach the low temperature rigid lattice *M*_2_ plateau prior to β-relaxation due to the experimental limitations. The distinct *M*_2_ step has only rarely been observed for thermosets (see Figure 4 in Reference [19]) consistent with the presence of significant distributions for β-relaxation dynamics. These limitations result in the poorly constrained fitting of the initial β-relaxation process, such that we will concentrate only on the larger α-relaxation (glass transition) region at higher temperatures.

### 2.4. Distributions in BTT-TCDDA Networks

**Figure 8:**
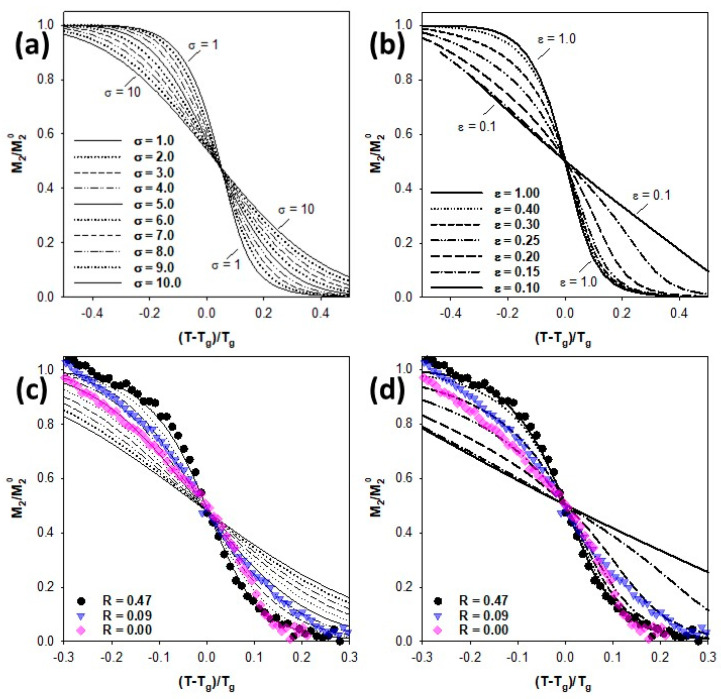
Reference curves for spectral second moment (*M*_2_) variation as a function of the distribution widths for (**a**) a Gaussian distribution in E_a_, (**b**) a Davidson-Cole distribution in correlation times, and (**c**,**d**) examples of estimating distribution widths for the BTT-TCDDA networks. Only selected R values are shown for clarity (see additional discussion in text). For the experimental data, the *M*_2_ was normalized by *M*_2_ = 540 kHz^2^ to reflect the partial averaging due to the β-relaxation dynamics present prior to the T_g_.

By considering the *M*_2_ variation near T_g_ and utilizing the reduced temperature, reference curves for the *M*_2_ variation with either a Gaussian or a DC distribution were developed, as shown in Figure 8a,b, respectively. With increasing distribution widths (increasing σ or decreasing ε), the *M*_2_ variation across the glass transition region is broadened. For the Gaussian distribution, only σ changes on the order of ±1 are distinguishable, while, for the DC distributions, ε variations on the order of ±0.05 can be distinguished, except between ε = 0.5 and 1.0, where the impact on the *M*_2_ variation is minimal. The overall *M*_2_ behavior in the reference curves is similar for the Gaussian and DC distribution models, but they become slightly more distinct for very large distribution widths. For the current study, the differences in the distribution widths as a function of the composition R are not large enough for the *M*_2_ analysis to reliably distinguish between the Gaussian and DC distribution models.

The *M*_2_ temperature variation for the BTT-TCDDA networks are plotted in Figure 8c,d on the Gaussian and DC distribution reference curves. There is clearly an increase in the distribution with the decreasing R, and assuming a Gaussian distribution, we can assign σ accordingly. Starting from σ < 1 for R = 0.47 and then increasing to σ = 2 for R = 0.19, σ = 3 to 4 for R = 0.09 and σ = 5 for R = 0.0. The *M*_2_ response for the R = 0.47 and R = 0.32 curves were not distinguishable and suggest that the distribution change between these two R compositions is within one sigma. Similarly, assuming a DC distribution, ε > 0.4 for the R = 0.47 and R = 0.32 compositions (recall that, between ε = 0.5 and 1.0, the reference curves are indistinguishable), then the distribution width increases with ε = 0.35 for R = 0.19, ε = 0.3 for R = 0.09, and to ε = 0.25 for the R = 0.0 network. It is also important to note that the *M*_2_ behavior for the R = 0.0 BTT-TCDDA network is not symmetric around T_g_, which we have attributed to the degradation of this material at the very high temperatures (> 600 K or > 325 °C) achieved in the variable temperature experiments for this composition, and produced irreversible changes in the *M*_2_ behaviors. These results are summarized in Table 1 and reveal that, in the BTT-TCDDA networks, the distribution in E_a_ (and, correspondingly, τ_c_) increases with the decreasing R. This is consistent with the picture of increased heterogeneous dynamics due to the chain-growth mechanism being dominant at high acrylate concentrations.

### 2.5. Dynamic Correlation Times

**Figure 9:**
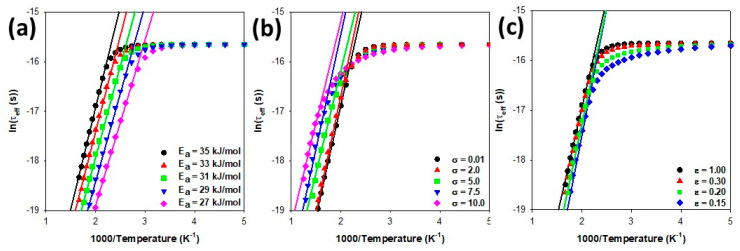
Simulated Arrhenius behavior of the effective correlation time (τ_eff_) obtained from the *M*_2_ using Equation (A6), assuming (**a**) different activation energies (E_a_) for the dynamic process (τ_0_ = 0.5905 ns, fixed), (**b**) assuming a $M_{2}^{Dist}$ (Equation (A7)) for a Gaussian E_a_ distribution with a mean E_a_ = 33 kJ/mol (Equation (A8)), and (**c**) assuming a $M_{2}^{Dist}$ for a Davidson-Cole distribution (Equation (A9)).

While the reference curves presented above allowed the distribution in activation energies or correlation times to be assessed, we also wanted to explore the impact distributions have on the Arrhenius analysis that had assumed a single correlation time when analyzing the *M*_2_ temperature behavior. An “effective” correlation time, τ_eff_, to describe the polymer chain fluctuations near the T_g_ was determined using Equation (A6) (additional discussion is provided in the Appendix A) [6,7,19,20,49]. Activation energies were then estimated assuming the Arrhenius behavior of τ_eff_ in the high temperature limit. The low temperature plateau shows an invariant τ_eff_ and is an artifact of the dynamics not being fast enough at these low temperatures to average the dipolar coupling (i.e., very slow motions are not probed by the ^1^H *M*_2_). For a Gaussian distribution of activation energies with small σ (Figure 9a), the linear Arrhenius behavior through the glass transition is easily fit, giving well-defined activation energies (± 0.5 kJ/mol). With the increasing σ, defining the linear region for analysis becomes more difficult. For example, assuming a Gaussian distribution with increasing σ (Figure 9b), the variation of τ_eff_ across the T_g_ shows more curvature. This is intuitively consistent, because there are both dynamic processes that can average the dipolar coupling active at lower temperatures (lower E_a_), as well as dynamics requiring higher temperatures (higher E_a_) to become active. The curvature in the τ_eff_ high-temperature behavior increases the error in measuring the E_a_ and can become as large as ± 5 kJ/mol (for the variation in distributions shown in Figure 9b), depending on the temperature range for which the linear regression is defined. The average E_a_ for a Gaussian distribution can be calculated from the temperature of the midpoint in the *M*_2_ transition regardless of σ (Figure 3a), if the pre-exponential τ_0_ factor is known and is invariant (see Section 2.6), but the *M*_2_ line shape analysis does not allow these two parameters to be distinguished [38].

Similarly, increasing the DC distribution also enhances the curvature of the τ_eff_ temperature behavior (Figure 9c), but is slightly less pronounced compared to the larger Gaussian distributions and can produce an uncertainty of ± 2 kJ/mol for the range of ε evaluated. Since the DC distribution is nonsymmetric, the average τ_c_ varies with the increasing ε and is responsible for the shifting of the midpoint in the *M*_2_ transition (Figure 4b). An inspection of Figure 9b,c shows that the difference in the τ_eff_ behavior between the Gaussian or DC distribution is subtle and would be difficult to distinguish experimentally.

**Figure 10:**
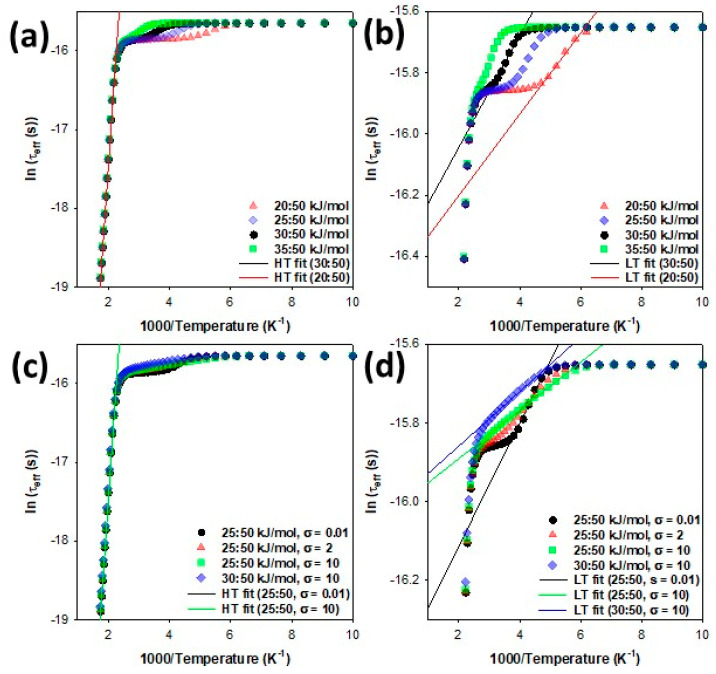
Simulated Arrhenius behavior of the effective correlation time (τ_eff_) (estimated using Equation (A6)) for two dynamic processes defined by the activations energies E_a_(1):E_a_(2), (**a**) assuming variations in the activation energies E_a_(1) of the first motion, (**b**) an expansion of the *M*_2_ transition for the first motion, (**c**) behavior assuming a Gaussian distribution of E_a_(1), and (**d**) an expansion of the *M*_2_ transition for the first dynamical process. HT and LT indicate high-temperature and low-temperature regions, respectively.

In the presence of multiple dynamic processes, the temperature behavior of the τ_eff_ increases in complexity, as explored in Figure 10. Recall that the τ_eff_ estimated using Equation (A6) assumes a single dynamic process. Inversion of the *M*_2_ variation in the presence of multiple dynamic processes (Equation (A10)) to define a single effective τ_eff_ is not possible, such that Equation (A6) is strictly valid only for a single motion. If the *M*_2_ transitions are well-separated (the dynamic motions have very different E_a_) and one considers each *M*_2_ transition separately, then it is possible to obtain more accurate E_a_ estimates. By estimating a single τ_eff_ for systems containing multiple dynamic processes, an incorrect estimate for the E_a_ describing the initial transition (β-relaxation) is observed for both the Gaussian and DC distributions results (Figure 9b,c) and should be avoided. If the entire β transition in the *M*_2_ could be experimentally measured, then it would be possible to correctly determine the E_a_(1). As noted previously, experimentally, our NMR probe limits us from reaching very low temperatures and prevents a complete analysis of the β transition. Figure 10a,c do reveal that using a τ_eff_ for the α-relaxation process allows the correct measurement of the E_a_(2) if the first dynamic process (i.e., β-relaxation) is well-separated (Ea > 40% different). Therefore, the activation energy corresponding to the glass transition temperature can be measured and will be the focus of the subsequent analysis for the BTT-TCDDA networks.

### 2.6. Arrhenius Behavior for TCDDA-BTT Networks

**Figure 11:**
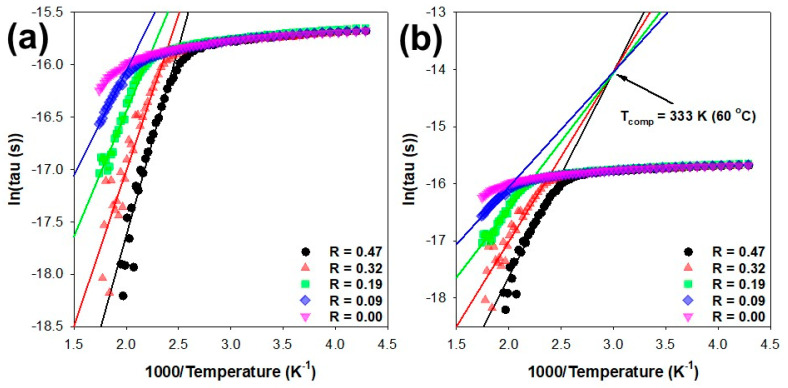
(**a**) Arrhenius behavior of the effective correlation time (τ_eff_) in the BTT-TCDDA networks as a function of R. The τ_eff_ was estimated using Equation (A6), and (**b**) the Arrhenius behavior showed the compensation temperature (T_comp_).

Using the insight from the discussion above, the Arrhenius behavior of τ_eff_ for the BTT-TCDDA networks for different compositions is shown in Figure 11 and summarized in Table 1. Only the high-temperature glass transition (α-relaxation) was evaluated, with the E_a_ for the R = 0.0 network not reported because of an insufficient number of high-temperature data points. The E_a_ values are ~2 times smaller than those we reported for epoxy thermosets [19] and reflect the lower T_g_ values in these thiol-acrylate networks. When decreasing R, the activation energy decreases from 29.2 to 16.7 kJ (even though the T_g_ is increasing), while the pre-exponential term (τ_0_) increases by a factor of 10 over the same R range (Table 1). Both E_a_ and τ_0_ reveal a linear dependence in the composition ratio (R) (Figure S4). The activation energy (E_a_) increases for networks with larger R, while the entropy of activation (ln(τ_0_)) decreases for larger R. This behavior may be related to differences in the free volume fraction of the BTT and TCDDA moieties and the relative contributions of each to the network free volume with changing R compositions. Similar trends in E_a_ and τ_0_ have been observed for the polymer relaxation in nano-patterned polymer films [50] and polymer thin films [51]. From the Arrhenius behavior of τ_eff_, a compensation or isokinetic temperature (T_comp_) was determined (Figure 11b) to be ~333 K (60 °C), which is below the T_g_ for all R compositions but falls near the cure temperature (25 °C). The compensation temperature corresponds to that temperature at which the chain dynamics are equivalent for all R networks.

**Figure 12:**
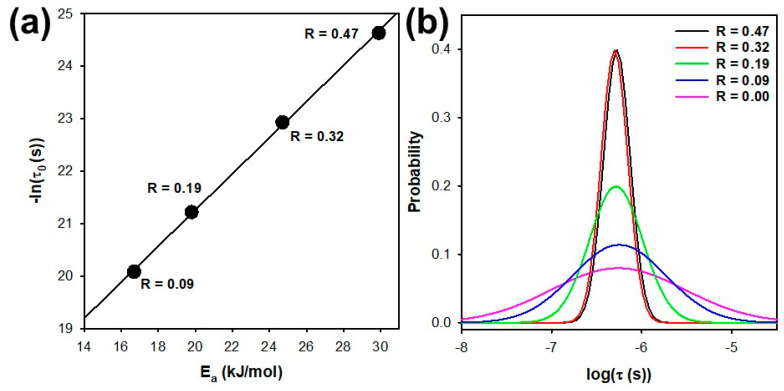
(**a**) Correlation between the measured correlation time pre-exponential factor (τ_0_) of the Arrhenius equation (Equation (2)) and the activation energy (E_a_) and (**b**) the τ_c_ distributions from the experimental results in Table 1 for the different BTT-TCDDA networks. For the R = 0.0 composition, E_a_ and τ_0_ were estimated from the linear dependence of these parameters with R, as shown in Figure S4.

A linear correlation between the pre-exponential factor and the activation energy is observed for these BTT-TCDDA networks (Figure 12a) and is an example of the compensation effect or enthalpy entropy compensation (EEC) [52]. The EEC phenomena is commonly reported in glass-forming polymer liquids [53,54,55]. The physical significance of compensation in polymers is still under discussion but has been related to the presence of cooperative dynamics during the glass transition [56,57], along with local structural heterogeneity and distributions of corresponding dynamic activation energies [58]. A complete analysis of the ECC effect is beyond the scope of this manuscript, but it is suggested that local dynamic heterogeneity plays a role in the ECC behavior in BTT-TCDDA networks.

Using Table 1, the distributions in the correlation times (τ_c_) for the different network compositions are shown in Figure 12b. These results support the notion that, during the cure of a homogeneous network, such as R = 0.47, the chain dynamics are very uniform, as the polymer chains are trapped in the glassy phase during sample vitrification. In contrast, during the cure of heterogeneous networks, such as R = 0.0, there is a distribution of chain dynamics where a population of mobile chains is retained as the material enters the glassy phase. This mobile fraction allows for residual functional groups to become spatially near each other due to chain diffusion, leading to additional crosslinking at a given cure temperature and, subsequently, higher T_g_ values. Continued crosslinking (and increasing T_g_) will shift the distribution of polymer chain dynamics to slower τ_c_ values, until there are no longer any mobile chain fragments available that would permit further crosslinking. The remaining R compositions are intermediate between these limiting networks.

## 3. Materials and Methods

### 3.1. NMR Spectroscopy

Wide-line solid-state ^1^H NMR spectra were acquired on a Bruker Avance III instrument operating at an observe frequency of 400.14 MHz using a 7-mm high-temperature DOTY MAS probe (DOTY Scientific Inc, Columbia, SC, USA) under static (non-spinning) conditions. All 1D static ^1^H NMR spectra used a Hahn echo (HE) pulse sequence with an inter-pulse delay of 10 μs, 16 scan averages, and a 5-s recycle delay. Variable temperature experiments were conducted between 233 K (−40 °C) and 673 K (+400 °C) using a 5-min temperature equilibration prior to acquisition. The second moment (*M*_2_) of the ^1^H NMR spectra was evaluated using Equation (1) using MATLAB (MathWorks, Inc., Natick, MA, USA). The NMR observed glass transition temperature T_g_(NMR) was obtained by fitting the *M*_2_ temperature variation using:

(3) $$M_{2}\left(T\right)=M_{2}^{\prime \prime }+\frac{M_{2}^{\prime }}{1+\exp\left[-\left(\frac{T-T_{g}\left(\mathrm{NMR}\right)}{b}\right)\right]}$$

where $M_{2}^{\prime }$ is the motionally averaged moment at temperatures just prior to the T_g_ transition, $M_{2}^{\prime \prime }$ is the residual second moment above the T_g_ transition, and 1/b is the rate of the temperature-induced change occurring at T_g_. The limits were chosen near T_g_ to separate the transition from the other motional (i.e., β-relaxation) *M*_2_ variations. The distribution analysis used reduced temperatures ((T-T_g_)/T_g_) incorporating T_g_(NMR) [19]. In general, T_g_(NMR) was found to be higher than the T_g_ determined from DMA analysis and reflects the NMR time probed through the averaging of the dipolar coupling.

### 3.2. Materials

The preparation of these polymer materials has recently been detailed [20]. Briefly, tricyclodecane dimethanol diacrylate (TCDDA) and the aromatic comonomer 1,3,5-benzenetrithiol (BTT) were mixed with different ratios of the reactive functional groups defined by the ratio R = (SH)_0_/(C=C)_0_, where (SH)_0_ and (C=C)_0_ were the initial concentrations of the thiol and acrylate functional groups, respectively. The photo initiator *p*-xylylene bis(*N,N*-diethyldithiocarbamate) (XDT) was added to the mixture at a 1 wt% concentration. The samples were photocured at room temperature (RT) using a Henkel (Düsseldorf, Germany) Loctite Zeta 7400 UV lamp. The light intensity was 100 mW/cm^2^ at 365 nm (300 mW/cm^2^ at 254 nm) measured using a Thorlabs (Newton, NJ, USA) PM100D power meter with a S120VC photodiode sensor. The sample temperature was observed to increase to ~80 °C during the UV cure. After photocuring, the samples were further thermally post-cured (in the absence of UV light) by heating to 250 °C at 3 °C/min.

## 4. Conclusions

The impact of distributions in activation energies and correlation times describing dynamic motions on the temperature behavior of the NMR spectral second moment, *M*_2_, were evaluated and allowed methods to be developed for the experimental measurement of distributions and activation energies. It was demonstrated that, with the use of a T_g_-reduced temperature scale, it is possible to directly compare the *M*_2_ behavior during the glass transition of polymers over a wide range of conditions. A set of reference curves were developed to address the impact of distribution widths, assuming a Gaussian distribution of activation energies or a Davidson-Cole distribution of dynamic correlation times. These reference curves were used to estimate changes in the distributions from the ^1^H NMR *M*_2_ temperature variations for a series of BTT-TCDDA networks. These NMR *M*_2_ analyses demonstrated that there is an increase in the distribution of dynamic relaxation rates for the polymer chain motion near T_g_ for networks dominated by chain-growth polymerization chemistry and that this leads to increasing T_g_ values at a given cure temperature.

## Figures and Tables

**Table 1:** Activation energies, pre-exponential constant, and distribution parameters for the glass transition in the 1,3,5-benzenetrithiol-tricyclodecane dimethanol diacrylate (BTT-TCCDA) networks obtained from the static ^1^H NMR second moment analysis.

Network	T_g_ (NMR) (K)^a^	E_a_ (kJ/mol)	τ_0_ (ns)	σ(E_a_)^b^	ε^c^
R = 0.47	412	29.9 ± 2	0.02 ± 0.03	<1	>0.4
R = 0.32	440	24.7 ± 2	0.11 ± 0.06	<1	>0.4
R = 0.19	482	19.8 ± 1	0.61 ± 0.1	2	0.35
R = 0.09	523	16.7 ± 1	1.9 ± 0.2	3.5	0.30
R = 0.00	560	n.d. ^d^	n.d. ^d^	5	0.25

^a^ Glass transition temperature estimated from the NMR second moment (*M*_2_) transition. ^b^ Gaussian distribution width in kJ/mol. ^c^ Davidson-Cole distribution parameter. ^d^ n.d. = not determined due to insufficient high temperature data. E_a_: activation energy. τ_0_: pre-exponential correlation time.

## Supplementary Materials

Supplementary materials can be found at https://www.mdpi.com/1422-0067/21/15/5176/s1: Figure S1: DMA analysis from the BTT-TCDDA networks. Figure S2: Simulated *M*_2_ behavior with variation in pre-exponential correlation time. Figure S3. Simulated probability distributions. Figure S4. Correlations between R and E_a_ or τ_0_.

## Abbreviations

MDPI	Multidisciplinary Digital Publishing Institute
BTT	1,3,5-benzenetrithiol
DC	Davidson-Cole
DMA	Dynamic mechanical analysis
DOAJ	Directory of open access journals
EEC	Entropy enthalpy compensation
*M*_2_	Second moment
NMR	Nuclear Magnetic Resonance
TCDDA	Tricyclodecane dimethonal diacrylate
T_g_	Glass transition temperature
T_g_(NMR)	Glass transition temperature obtained from NMR *M*_2_ analysis
UV	Ultraviolet

## Appendix A

### A.1. NMR Second Moment

The second moment ($M_{2}$) of the NMR spectral line shape f(ω) as a function of frequency ω, around a maximum at frequency $\omega_{0}$, is defined by [6,7]

(A1)

For static NMR spectra whose line width is dominated by dipolar couplings, $M_{2}$ was first derived by Van Vleck [59], with the impact of molecular reorientation further described by Powles and Gutowsky [49,60]:

(A2)

For *N* identical nuclei, $M_{2}$ is the average over the summation of the *N(N-1)* dipolar interactions [45]:

(A3)

In the presence of dynamics, the pair-wise moments are related to the spectral density describing the correlation function of the reorientation, where the line width ±δν defines the frequencies impacting the *M*_2_ [41,42,45,60]:

(A4)

For a single dynamic process, the second moment is a function of the motional correlation time and can be defined by [49,60,61]

(A5)

where $M_{2}^{i}$ is the second moment at a given temperature *i*, $M_{2}^{0}$ is the rigid lattice limit of the second moment, 〈$M_{2}$〉 is the completely motionally averaged second moment for that dynamic process, and $\delta_{\omega }=2\omega \delta \nu =\sqrt{M_{2}}$. The correlation time at a given temperature is directly obtained from Equation (A5).

(A6)

Here, we have denoted it as an effective correlation time to reflect the possibility of distributions and multiple dynamic processes, as discussed in the Results section. Equation (A6) is equivalent to the correlation time we used previously based on the square of the line widths [19].

### A.2. Correlation Time Distributions

It was also important to consider the presence of correlation time distributions to realistically describe the polymer dynamics [62]. One can define the probability distribution of the correlation times as $P(\tau_{c})$, allowing the calculation of the average second moment using

(A7) $$\begin{array}{l} M_{2}^{Dist}=\langle M_{2}\rangle +\left(M_{2}^{0}-\langle M_{2}\rangle \right)\frac{2}{\pi }\int_{0}^{\infty }P\left(\tau_{c}\right)\tan^{-1}\left(\sqrt{M_{2}}\tau_{c}\right)d\tau_{c}\\ \mathrm{where}\;\int_{0}^{\infty }P\left(\tau_{c}\right)\;d\tau_{c}=1 \end{array}$$

Many different distribution functions have been developed to describe dynamics in solids [37,62]. The distributions are commonly formulated using the dimensionless reduced parameter $z=\ln(\tau_{c}=/\tau_{c}^{*})$, where $\tau_{c}^{*}$ is a characteristic correlation time, such as the limit or center correlation time of the distribution. For example, a Gaussian distribution of the activation energies (E_a_) gives rise to the log-Gaussian distribution of $\tau_{c}$

(A8)

where $\ln\tau_{c}$ is the log mean correlation time and *σ* is the log of the standard deviation. An equivalent Gaussian expression involving a distribution in the E_a_ can also be used. The Davidson-Cole (DC) distribution [39] is another function commonly used to interpret dynamics in polymers and glasses and is defined by [39,62]

(A9)

with $z=\ln(\tau_{c}=/\tau_{c}^{0})$, $\tau_{c}^{0}$ is the maximum correlation time in the distribution, with an Arrhenius temperature dependence, and *ε* is dimensionless parameter that specifies the breadth of the DC distribution. The DC model incorporates the idea of defect regions within the network that have lower E_a_ with correlation times that are smaller than $\tau_{c}^{0}$, the characteristic correlation time of chain dynamics, while slower motions (larger E_a_) are completely missing or quenched.

### A.3. Internal Dynamic Impact on the Second Moment

The variations of the NMR line shape *M*_2_ due to averaging from multiple, complex internal dynamics have been described by a variety of different groups [40,41,42,43,44,45,46,47,48] and have included combinations of methyl tunneling and jump dynamics, 2-site and 3-site motions, and 3-site jumps plus and molecular reorientation, as well as diffusion across lattice sites. The temperature variation of the second moment *M*_2_ in the presence of two uncorrelated dynamics, leading to the dipolar averaging, is described by [41]

(A10)

with

(A11)

where $M_{2}^{0}$ is the second moment of the NMR spectral line shape in the rigid lattice, $\bar{M_{2}}$ is the second moment of the average NMR line shape due to the first dynamic process (e.g., local molecular fluctuations of the β-relaxation process), $\overset{=}{M_{2}}$ is the second moment of the averaged line shape due to the second dynamical process (*e.g.,* polymer chain fluctuations of the α-relaxation glass transition temperature), and 〈$M_{2}$〉 is the NMR line shape averaged by both the first and second dynamic processes. The correlation times for the first and second processes are given by $\tau_{c1}$ and $\tau_{c2}$, respectively.
