# Supplementary material for: Heterogeneous Polymer Dynamics Explored Using Static 1H NMR Spectra
Source: Int J Mol Sci. 2020 Jul 22;21(15):5176. doi: 10.3390/ijms21155176 (PMC7432559; doi:10.3390/ijms21155176)
Supplement: Supplementary file 1 [file ijms-21-05176-s001.pdf]

Supporting Information

Heterogeneous Polymer Dynamics Explored Using Static ^{1}H NMR Spectra

Todd M. Alam, Joshua P. Allers and Brad H. Jones

Sandia National Laboratories, Department of Organic Materials Science
Albuquerque, NM 87185

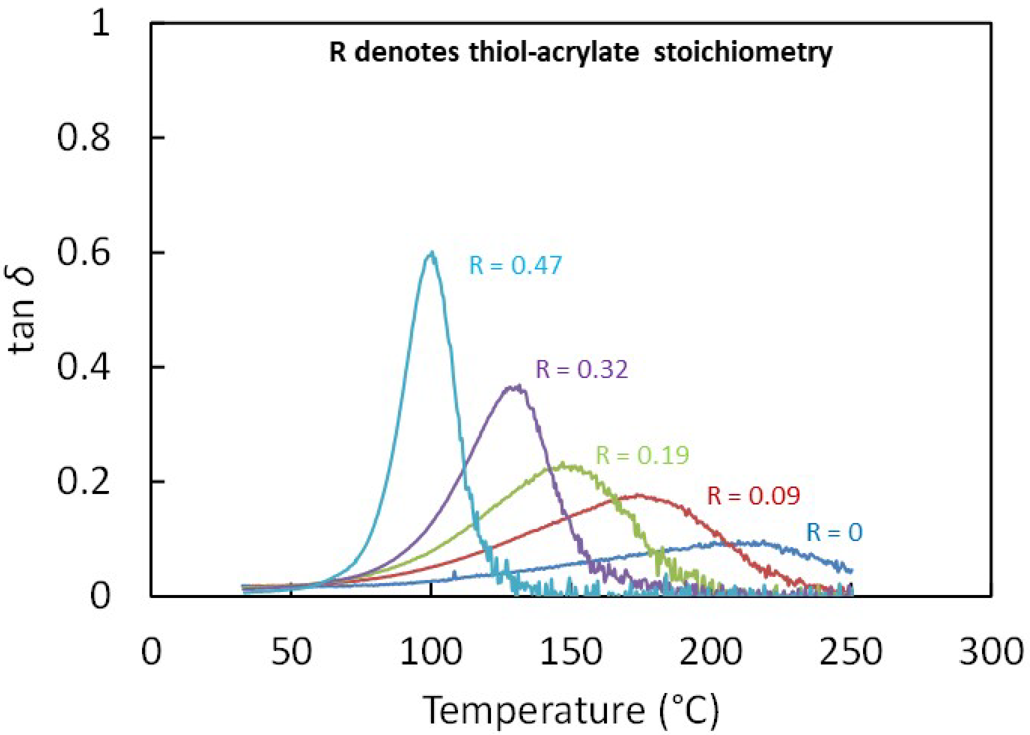

Figure S1. Loss tangent versus temperature obtained from dynamic mechanical analysis (DMA) for fully cured BTT-TCDDA networks with different thiol-acrylate stoichiometry (R = $(SH)_0/(C=C)_0$). With decreasing R, T_g and the breadth of the transition increases.

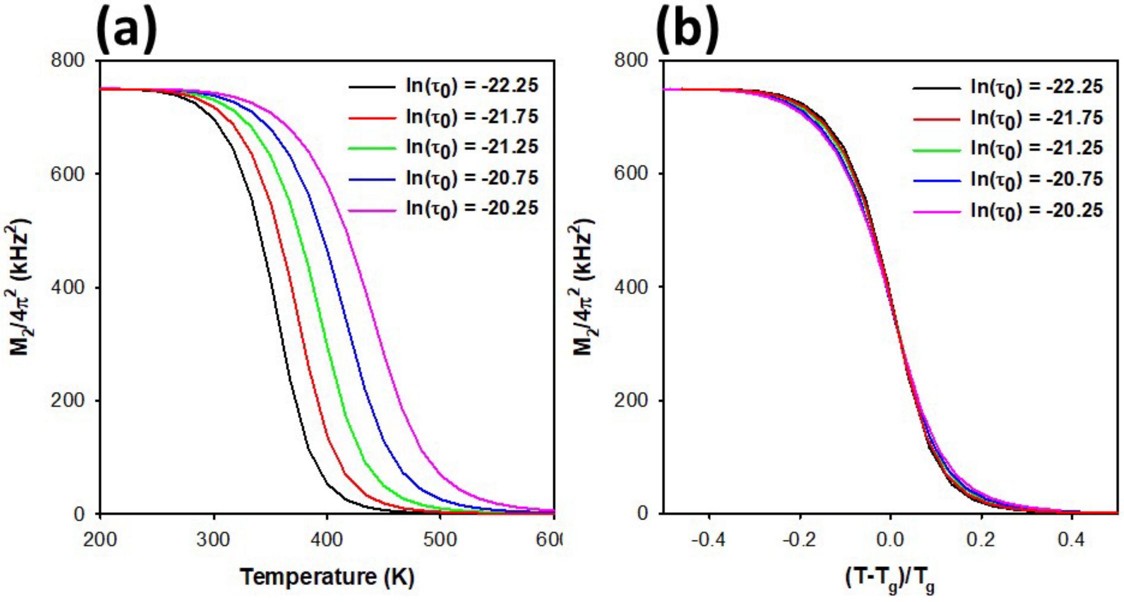

Figure S2. Simulation of the variation of the spectral second moment (M_2) for different pre-exponential correlation time, τ_0, assuming the Arrhenius temperate dependence shown in Eqn. 2 as a function of **(a)** temperature and **(b)** the reduced temperature. The activation energy, E_a, was held constant at 33 kJ/mol.

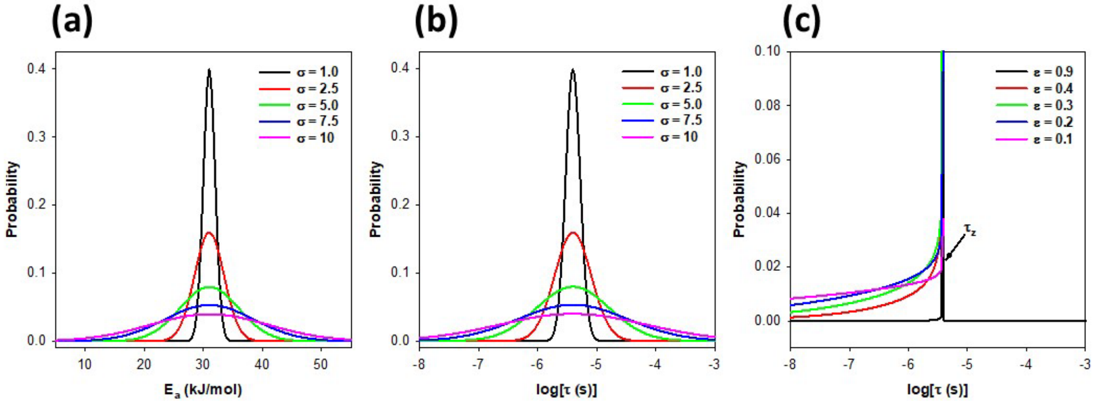

Figure S3. Simulated probability distribution assuming (a) a Gaussian distribution (Eqn. A8) of the activation energy E_a for different distribution widths σ (b) the corresponding correlation time τ distribution using Arrhenius relationship (Eqn. 2), and (c) a Davidson-Cole distribution with the characteristic correlation time τ_z and the distribution parameter ε (Eqn. A9). Simulations obtained assuming a mean E_a = 31 kJ/mol, τ_0 = 0.5905 ns and T = 423 K.

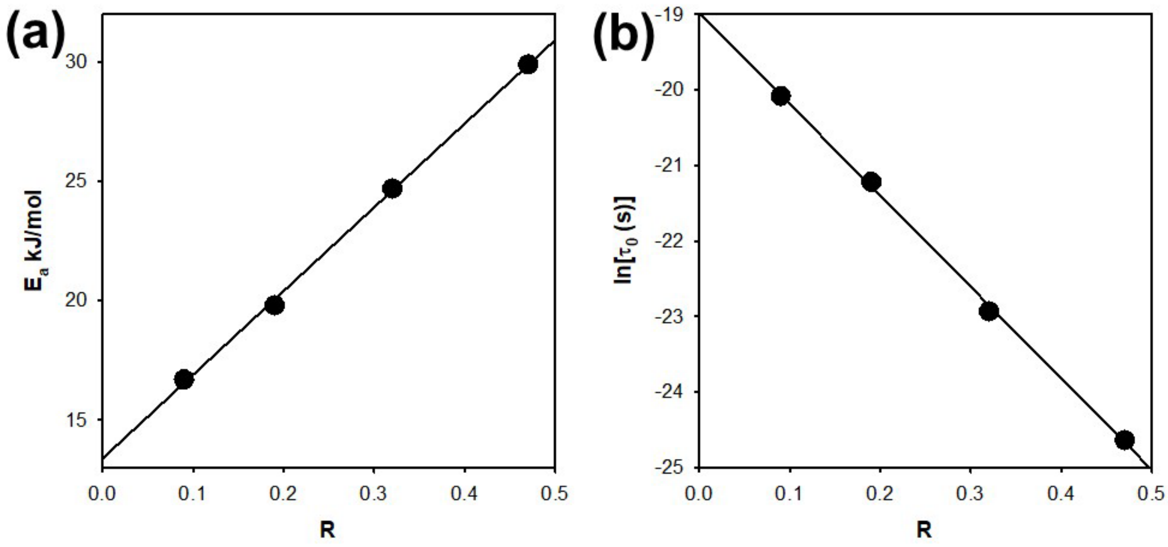

Figure S4. Correlation between thiol-acrylate stoichiometry (R = $(SH)_0/(C{=}C)_0$) and **(a)** activation energy (E_a) and **(b)** the natural log of the pre-exponential correlation time (τ_0) obtained from the analysis of the Arrhenius temperature behavior in **Figure 11**.
